# Serpine 1 induces alveolar type II cell senescence through activating p53‐p21‐Rb pathway in fibrotic lung disease

**DOI:** 10.1111/acel.12643

**Published:** 2017-07-19

**Authors:** Chunsun Jiang, Gang Liu, Tracy Luckhardt, Veena Antony, Yong Zhou, A. Brent Carter, Victor J. Thannickal, Rui‐Ming Liu

**Affiliations:** ^1^ Division of Pulmonary, Allergy, and Critical Care Department of Medicine School of Medicine University of Alabama at Birmingham Birmingham AL USA

**Keywords:** aging, lung fibrosis, p53, serpin 1, type 2 alveolar epithelial cell senescence

## Abstract

Senescence of alveolar type 2 (ATII) cells, progenitors of the alveolar epithelium, is implicated in the pathogeneses of idiopathic pulmonary fibrosis (IPF), an aging‐related progressive fatal lung disorder with unknown etiology. The mechanism underlying ATII cell senescence in fibrotic lung diseases, however, remains poorly understood. In this study, we report that ATII cells in IPF lungs express higher levels of serpine 1, also known as plasminogen activator inhibitor 1 (PAI‐1), and cell senescence markers p21 and p16, compared to ATII cells in control lungs. Silencing PAI‐1 or inhibition of PAI‐1 activity in cultured rat ATII (L2) cells leads to decreases in p53 serine 18 phosphorylation (p53^S18P^), p53 and p21 protein expressions; an increase in retinoblastoma protein phosphorylation (ppRb); and a reduction in the sensitivity to bleomycin‐ and doxorubicin‐induced senescence. Silencing p53, on the other hand, abrogates PAI‐1 protein‐stimulated p21 expression and cell senescence. *In vivo* studies, using ATII cell‐specific PAI‐1 conditional knockout mouse model generated recently in this laboratory, further support the role of PAI‐1 in the activation of p53‐p21‐Rb cell cycle repression pathway, ATII cell senescence, and lung fibrosis induced by bleomycin. This study reveals a novel function of PAI‐1 in regulation of cell cycle and suggests that elevation of PAI‐1 contributes importantly to ATII cell senescence in fibrotic lung diseases.

## Introduction

Cellular senescence, a state of permanent inhibition of cell growth, has been linked to aging and aging‐related diseases (Akram *et al*., 2014). The mechanisms underlying cellular senescence under either physiological or pathological conditions, however, remain poorly understood. Alveolar type II (ATII) cells can self‐renew and also differentiate into type I alveolar epithelial cells and therefore are considered as alveolar progenitor cells (Aoshiba *et al*., 2003, 2013). ATII cell senescence is evident in fibrotic lung diseases, including IPF (Buckbinder *et al*., 1995; Chang *et al*., 2010; Bhandary *et al*., 2013; Barkauskas & Noble, 2014), and in experimental fibrosis models (Chao, 2015; Childs *et al*., 2015). A current disease paradigm is that lung fibrosis develops as a result of unremitting insults plus genetic and aging‐related risk factors, leading to alveolar epithelial cell injuries, which is followed by activation of myofibroblasts and replacement of injured alveolar epithelium with fibrotic tissue, due to a decreased reparative capacity of alveolar epithelium. Elucidation of the mechanisms underlying ATII cell senescence, therefore, may be a key to our understanding of the disease pathogenesis and thus development of effective therapeutics.

Plasminogen activator inhibitor 1 (PAI‐1), also known as serpine 1, is a primary inhibitor of tissue type and urokinase type plasminogen activators (tPA and uPA, respectively), which convert plasminogen into plasmin, a serine proteinase playing a major role in fibrinolysis. Besides suppression of fibrinolysis, PAI‐1 has many other functions, including modulation of cell adhesion, migration, and proliferation, dependent or independent of its protease inhibitory activity (Chilosi *et al*., 2013). Studies from this laboratory and from others have shown that PAI‐1 plays a critical role in the development of lung fibrosis, although the mechanism whereby PAI‐1 promotes lung fibrosis remains elusive (El‐Deiry *et al*., 1992; Dimri *et al*., 1995; Citrin *et al*., 2013; Disayabutr *et al*., 2016). Importantly, PAI‐1 expression is increased in senescent cells (Elzi *et al*., 2012) and emerging evidence suggests that PAI‐1 is not merely a marker but also a mediator of cell senescence (Fernandez Perez *et al*., 2010; Eren *et al*., 2014a,b; Ghosh *et al*., 2016). Nonetheless, whether increased PAI‐1 expression is responsible for ATII cell senescence in fibrotic lung diseases and, most importantly, how PAI‐1 promotes cell senescence remain unclear.

Using IPF lung tissues, cultured ATII cells, and a tamoxifen‐inducible, ATII cell‐specific PAI‐1 conditional knockout mouse model generated recently in this laboratory, we show, in this study, that PAI‐1 induces p53, activates p53‐p21‐Rb cell cycle repression pathway, and mediates bleomycin‐ and doxorubicin‐induced ATII cell senescence both *in vitro* and *in vivo*. Suppression of ATII senescence by knocking out the PAI‐1 gene is associated with an attenuation of lung fibrosis. These results reveal a novel mechanism whereby PAI‐1 regulates cell cycle and suggest that elevated PAI‐1 contributes to ATII cell senescence in fibrotic lung diseases.

## Results

### ATII cells in IPF lungs express higher level of PAI‐1 and cell senescence markers p21 and p16

ATII cell senescence is evident in IPF lung. As PAI‐1 plays a critical role in cell senescence and in the development of lung fibrosis, we first examined whether senescent ATII cells in IPF lung express higher level of PAI‐1. Our results confirm that, compared to ATII cells in control lungs, ATII cells in IPF lungs express higher levels of PAI‐1 (Hogan *et al*., 2014) as well as p21 and p16 (Chang *et al*., 2010; Barkauskas & Noble, 2014), two‐cell senescence mediators. These results suggest that increased PAI‐1 expression may contribute to ATII cell senescence in IPF.

### Silencing PAI‐1 with PAI‐1 siRNA/shRNA reduces p53 and p21 expressions, increases Rb protein phosphorylation, and attenuates bleomycin‐induced ATII cell senescence

**Figure 1:**
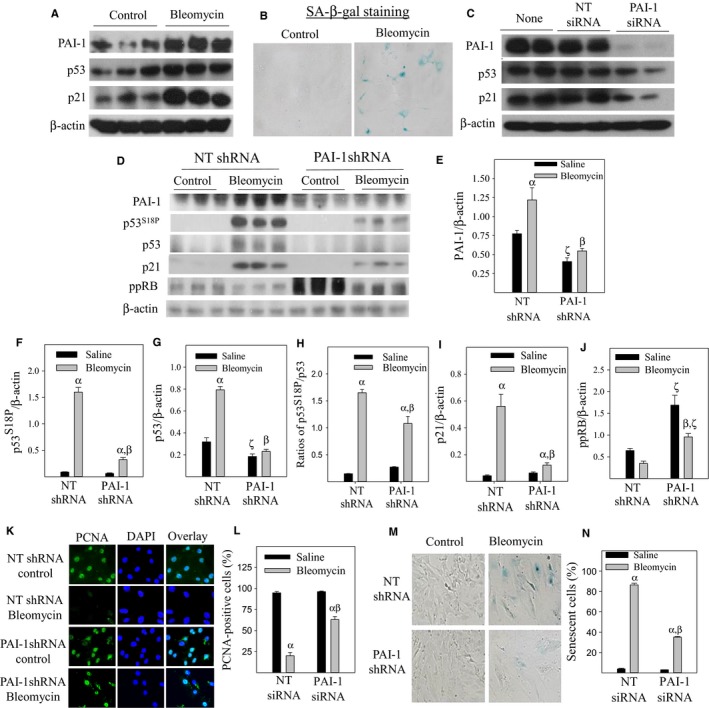
Knockdown of PAI‐1 with PAI‐1 siRNA/shRNA reduces p53 and p21 protein levels, increases Rb phosphorylation, and attenuates bleomycin‐induced L2 cell senescence. Rat ATII (L2) cells were treated with 50 mU/mL bleomycin for 24 h (A & B) and then cultured in bleomycin‐free medium for additional 72 h (B). (C) L2 cell was transfected with PAI‐1 siRNA or nontarget siRNA (NT siRNA). D‐N) PAI‐1 shRNA or NT shRNA stably transfected L2 cells were treated with bleomycin for 24 h and then cultured in bleomycin‐free medium for additional 24 (D–L) or 72 (M and N) hours. PAI‐1, serine‐18 phosphorylated p53 (p53^S−18P^), p53, p21, and phosphorylated Rb (ppRb) proteins were determined by Westerns. β‐Actin is used as loading control. D, representative Western blotting pictures; E‐J, semi‐quantified band intensities normalized by β‐actin. (K and L) Immunostaining and quantification of proliferating cell nuclear antigen (PCNA). (M and N) SA‐β‐gal activity revealed by X‐gal staining. α, Significantly different from corresponding saline‐treated cells; β, significantly different from bleomycin‐treated NT shRNA‐transfected cells; ζ, significantly different from corresponding NT shRNA‐transfected cells (*P* < 0.05, *n* = 3–5).

Although PAI‐1 expression is increased in ATII cells in IPF lung, whether increased PAI‐1 is responsible for, and how PAI‐1 induces, ATII cell senescence are unknown. p53 is a master cell cycle regulator, which induces cell cycle arrest or senescence mainly through inducing p21, a cyclin‐dependent kinase inhibitor. p53 expression is increased in ATII cells in IPF lung (Buckbinder *et al*., 1995; Barkauskas & Noble, 2014). To determine whether increased PAI‐1 expression is responsible for ATII cell senescence in fibrotic lungs and whether PAI‐1 induces ATII cell senescence through increasing p53, rat ATII (L2) cells were treated with bleomycin. The results show that treatment of L2 cells with bleomycin significantly increases the protein levels of PAI‐1, p53, and p21 as well as the activity of senescence associated beta‐galactosidase (SA‐β‐gal), a marker of cell senescence (Fig. 1A,B). Silencing PAI‐1 with PAI‐1 siRNA, on the other hand, dramatically reduces the basal levels of p53 and p21 proteins in L2 cells (Fig. 1C). These results indicate that PAI‐1 positively regulates p53 and p21 expression and may mediate bleomycin‐induced ATII cell senescence through activating p53‐p21 pathway.

**Figure 2:**
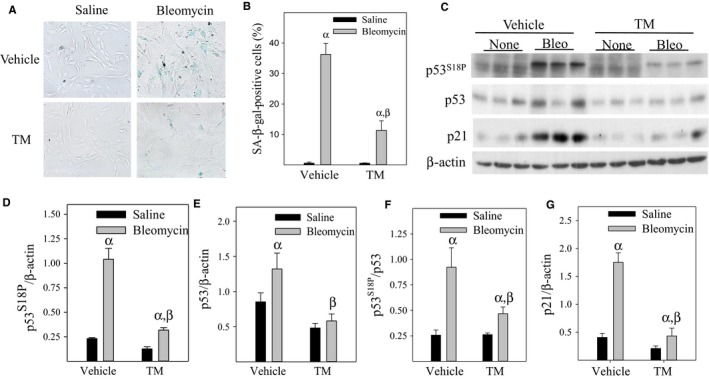
Inhibition of PAI‐1 activity with a small molecule PAI‐1 inhibitor TM5275 attenuates bleomycin‐induced L2 cell senescence. L2 cells were treated with 50 mU/mL bleomycin in the presence or absence of 25 μm of TM5275 for 24 hours and then cultured in bleomycin‐free medium for additional 72 (A and B) or 24 (C–G) hours. (A and B) SA‐β‐gal activity was revealed by X‐gal staining; (C–G) Western analyses of the proteins of interested in cell lysates, the band intensities semi‐quantified by ImageJ software, and normalized by β‐actin. α, Significantly different from corresponding saline‐treated cells; β, significantly different from bleomycin‐treated vehicle controls (*P* < 0.05, *n* = 3).

To further delineate the role of PAI‐1 in ATII cell senescence, L2 cells were stably transfected with PAI‐1 shRNA or nontarget shRNA (NT shRNA, control) and then treated with bleomycin. Western blotting results show that silencing PAI‐1 with PAI‐1 shRNA significantly increases the basal level of phosphorylated retinoblastoma protein (ppRb), attenuates bleomycin‐mediated increases in p53 serine 18 phosphorylation (p53^S18P^), p53 and p21 protein expression, and partially restores bleomycin‐mediated suppression of ppRb (Fig. 1D,J). The ratio of p53^S18P^ to p53 is significant increased with bleomycin treatment; silencing PAI‐1, however, attenuates bleomycin‐stimulated increase in the ratio (Fig. 2D,H), suggesting that PAI‐1 stimulates p53 phosphorylation at serine 18, a critical phosphorylation site for the stability of p53 protein (Huang *et al*., 2012, 2015), independence of its effect on p53 protein abundance. Associated with inhibition of p53 and p21 expression as well as stimulation of Rb phosphorylation, PAI‐1 shRNA ameliorates bleomycin‐mediated suppression of proliferating cell nuclear antigen (PCNA) expression (Fig. 1K,L) and increase in SA‐β‐gal activity (Fig. 1M,N). Together, the results suggest that PAI‐1 plays an important role in bleomycin‐induced ATII cell senescence and that PAI‐1 promotes ATII cell senescence at least in part by increasing p53 and thus p21, leading to a suppression of Rb protein phosphorylation.

### Inhibition of PAI‐1 activity with a small molecular PAI‐1 inhibitor TM5275 attenuates bleomycin‐induced p53 expression and L2 cell senescence

TM5275 is a small molecule PAI‐1 inhibitor, which, we have shown previously, blocks lung fibrosis in a bleomycin‐induced lung injury model (Disayabutr *et al*., 2016). To determine whether inhibition of PAI‐1 activity with TM5275 also protects ATII cells from bleomycin‐induced senescence, L2 cells were treated with 50 mU of bleomycin in the presence or absence of TM5275 (25 μm) for 24 h and then cultured in bleomycin‐free medium for additional 72 h. The results show that treatment of L2 cells with TM5275 significantly reduces bleomycin‐stimulated p53^S18^ phosphorylation, p53 and p21 expressions, as well as SA‐β‐gal activity (Fig.**** 2A–G). Our data also show that TM5275 reduces bleomycin‐stimulation increase in the ratios of p53^S18P^ to p53 (Fig. 2C,2F), further supporting the notion that PAI‐1 stimulates p53 serine 18 phosphorylation.

### Silencing PAI‐1 with PAI‐1 shRNA suppresses doxorubicin‐induced L2 cell senescence

**Figure 3:**
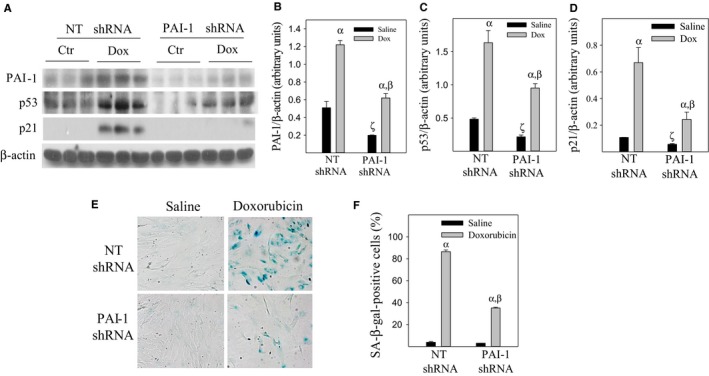
Knockdown of PAI‐1 protein with PAI‐1 shRNA attenuates doxorubicin‐induced L2 cell senescence. PAI‐1 shRNA or NT shRNA stably transfected L2 cells were treated with 50 nm of doxorubicin (Dox)/saline for 24 h and cultured in doxorubicin‐free medium for additional 24 h (A–D) or 72 h (E and F). PAI‐1, p53, p21, and β‐actin in cell lysates were determined by Westerns. A, representative Western blotting pictures; B–D, semi‐quantified band intensities by ImageJ program and normalized by β‐actin. E and F, SA‐β‐gal activity was revealed by X‐gal staining. α, Significantly different from the corresponding saline‐treated cells; β, significantly different from doxorubicin‐treated NT shRNA‐transfected cells; ζ, significantly different from saline‐treated NT shRNA‐transfected cells (*P* < 0.05, *n* = 3–5).

Doxorubicin (Dox) is another anticancer drug which induces senescence in different types of cells (Ghosh *et al*., 2016). Although the major toxicity of doxorubicin is in cardiovascular system, several studies have shown that doxorubicin therapy causes interstitial pneumonia and fibrosis in patients (Junqueira *et al*., 1979; Kunz *et al*., 1995; Kortlever *et al*., 2006). To determine whether doxorubicin induces ATII cell senescence and the role of PAI‐1 in this process, L2 cells that have been stably transfected with PAI‐1 shRNA or NT shRNA were treated with doxorubicin or saline. Similar to PAI‐1 siRNA (Fig. 1C), silencing PAI‐1 with PAI‐1 shRNA decreases the basal levels of p53 protein in L2 cells (Fig. 3A,C). Silencing PAI‐1 also diminishes doxorubicin‐induced p53 and p21 expressions (Fig. 3A–D) as well as SA‐β‐gal activity (Fig. 3E,F). The results suggest that doxorubicin induces ATII cell senescence and that PAI‐1 mediates doxorubicin‐induced ATII cell senescence at least in part by increasing p53.

### Silencing p53 abrogates PAI‐1 protein‐induced L2 cell senescence

**Figure 4:**
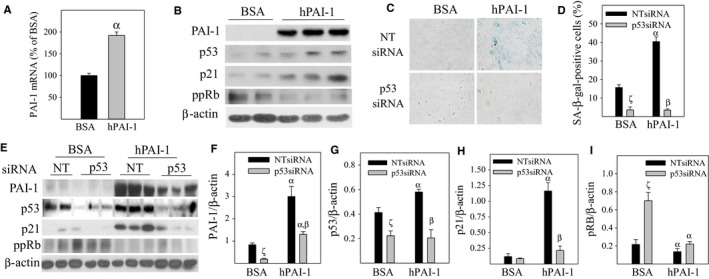
Knockdown of p53 protein with p53 siRNA abrogates PAI‐1 protein‐mediated L2 cell senescence. (A and B) L2 cells were treated with 1 μg/mL of hPAI‐1, dissolved in 0.1% BAS, or 0.1% bovine serum albumin (BSA) for 72 h. (A) PAI‐1 mRNA was determined by real‐time PCR; (B) Proteins of interest were determined by Westerns. (C–I) L2 cells were transfected with p53 siRNA or nontarget siRNA (NT siRNA) and then treated with hPAI‐1 or BSA for 72 h. (C and D) SA‐β‐gal activity was measured by X‐gal staining. (E–I) Western analyses of proteins of interest; E, representative Western blotting pictures; F–I, semi‐quantified band intensities normalized by β‐actin. α, Significantly different from corresponding 0.1% BSA (solvent) controls; β, significantly different from hPAI‐1‐treated NTsiRNA‐transfected cells; ζ, significantly different from BSA‐treated NTsiRNA‐transfected cells (*P* < 0.05, *n* = 3–5).

To further delineate the cause–effect relation between PAI‐1 and p53 in ATII cell senescence, L2 cells were transfected with p53 siRNA or nontarget siRNA and then treated with active human PAI‐1 protein (hPAI‐1, Molecular Innovation). The results show that treatment of L2 cells with hPAI‐1 increases PAI‐1 mRNA (Fig. 4A) as well as p53 and p21 proteins, which is associated with an suppression of Rb protein phosphorylation (Fig. 4B) and an increase in SA‐β‐gal activity (Fig. 4C,D). These data confirm that PAI‐1 activates p53‐p21‐Rb pathway and induces ATII cell senescence. The results also suggest that PAI‐1 induces the expression of its own gene (autocrine function). Silencing p53 with p53 siRNA, on the other hand, reduces the basal level of PAI‐1 protein and increases the basal level of Rb phosphorylation (Fig. 4E–I). These results suggest that p53 positively regulates PAI‐1 expression in ATII cells and that there is a feedforward relation between p53 and PAI‐1. Importantly, silencing p53 significantly reduces hPAI‐1‐mediated increases in p21 protein expression (Fig. 4E,H) and completely blocks hPAI‐1‐induced SA‐β‐gal activity (Fig. 4C,D). These results indicate that p53 functions as a downstream effector in PAI‐1‐induced ATII cell senescence, although it also regulates PAI‐1 expression.

### Deletion of PAI‐1 specifically in ATII cells in mice protects ATII cells from bleomycin‐induced senescence *in vivo*

To determine whether increased PAI‐1 is responsible for ATII cell senescence in fibrotic lung disease *in vivo*, a tamoxifen (Tmx) inducible, ATII cell‐specific PAI‐1 conditional knockout (Sftpc‐CreER:PAI‐1^fl/fl^; CKO) mouse model has been generated by gene targeting technique as deciphered in Fig. S1 (Supporting information). PCR results show that Tmx injection induces a deletion of the exons 4&5 in the PAI‐1 gene specifically in CKO mice (Fig. S2A, Supporting information). Immunofluorescence and Western analyses further confirm that Tmx injection almost completely knocks out PAI‐1 protein in ATII cells in CKO mice (Fig. S2B,C, Supporting information). The results suggest that we have successfully generated tamoxifen‐inducible, ATII cell‐specific PAI‐1 conditional knockout mouse model.

**Figure 5:**
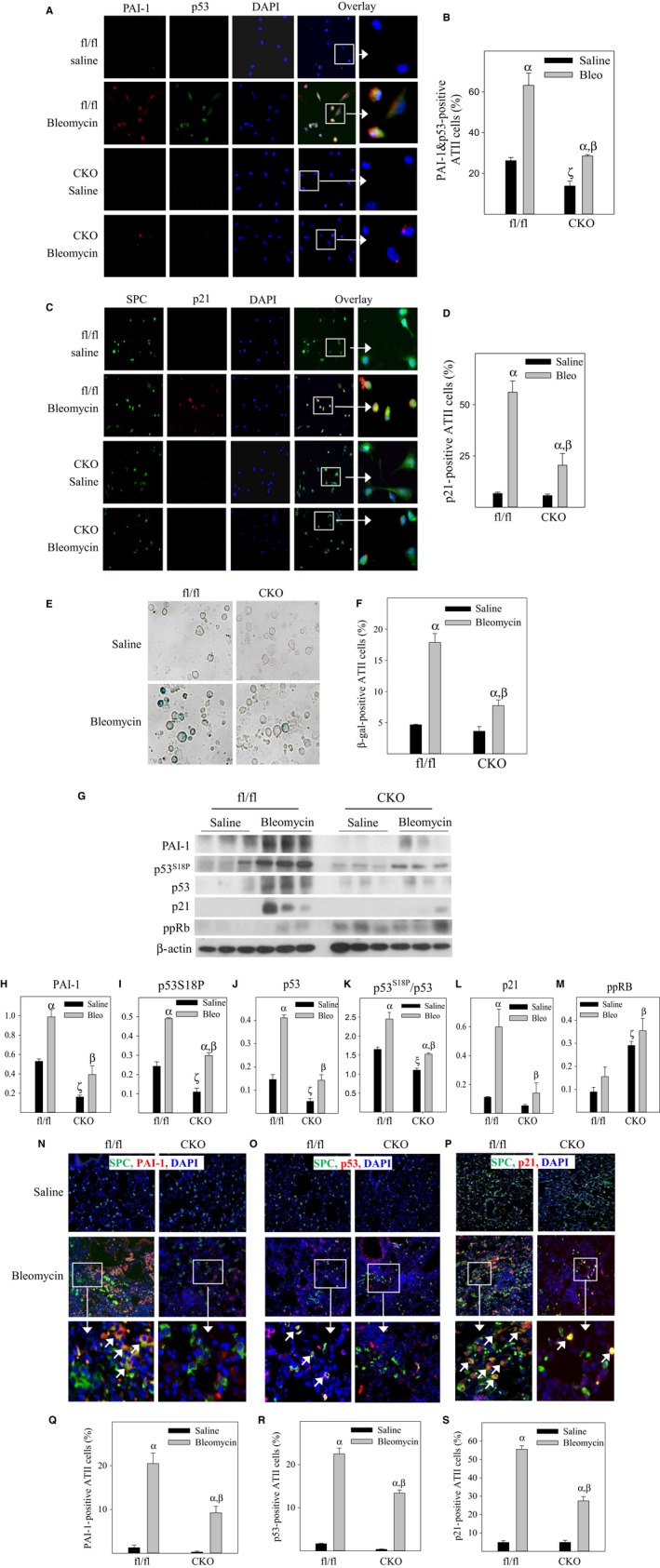
Knockout of the PAI‐1 gene specifically in ATII cells in mice attenuates bleomycin‐induced ATII cell senescence *in vivo*. (A and B) Double immunostaining of isolated ATII cells with anti‐PAI‐1 and anti‐p53 antibodies. (C and D) Double immunostaining of isolated ATII cells with anti‐p21 and anti‐SPC antibodies. (E and F) SA‐β‐gal activity in freshly isolated mouse ATII cells was revealed by X‐gal staining. Left panels are representative SA‐β‐gal staining pictures; right panel is quantitative data. (G–M) Western analyses of the proteins of interest in isolated ATII cells. (N–S) Double‐immunofluorescence staining of mouse lung tissues with PAI‐1, p53, or p21 and ATII cell marker SPC. Top panels are representative Western blotting pictures, and bottom panels are quantitative data. α, Significantly different from same genotype, saline‐treated mice; β, significantly different from bleomycin‐treated PAI‐1^fl/fl^ mice; ζ, significantly different from saline‐treated PAI‐1^fl/fl^ mice (*P* < 0.05, *n* = 3–6).

To test whether deletion of PAI‐1 specifically in ATII cells in mice will protect ATII cells from bleomycin‐induced senescence *in vivo* (Leung *et al*., 2015), CKO and wild‐type (PAI‐1 ^fl/f^) mice were intraperitoneally injected with tamoxifen and then challenged with 2 U/kg bleomycin. Mice were euthanized 14 days after challenge, lung tissue collected, and ATII cells isolated. Double‐immunofluorescence staining and X‐gal staining results show that bleomycin challenge significantly increases the numbers of ATII cells positive for PAI‐1 and p53 (Fig. 5A,B), for p21 (Fig. 5C,D), or for SA‐β‐gal activity (Fig. 5E,F) in wild‐type (PAI‐1^fl/fl^) mice. Deletion of PAI‐1 specifically in ATII cells in mice, on the other hand, significantly reduces bleomycin‐stimulated increases in PAI‐1/p53‐, p21‐, or SA‐β‐gal‐positive ATII cells (Fig. 5A–F). Western analyses with isolated ATII cells further show that deletion of PAI‐1 specifically in ATII cells in mice attenuates bleomycin‐stimulated increases in p53^S18^ phosphorylation (Fig. 5G,I) as well as the expression of p53 and p21 proteins (Fig. 5G,J,L). The ratios of p53^S18P^ to p53 is significantly increased following bleomycin treatment and returned to control level with deletion of PAI‐1 (Fig. 5G,K), suggesting that PAI‐1 stimulates p53^S18^ phosphorylation *in vivo* as well. Consistent with the results from cultured L2 cells, deletion of PAI‐1 alone increases Rb phosphorylation in ATII cells in mice (Fig. 5G,M). The effects of PAI‐1 deletion on bleomycin‐induced p53 and p21 expressions in ATII cells are also confirmed by double immunostaining of mouse lung tissues (Fig. 5N–S). These results provide strong evidence, for the first time, that increased PAI‐1 mediates bleomycin‐induced p53 expression and ATII cell senescence in lung fibrosis *in vivo*.

### Deletion of PAI‐1 specifically in ATII cells in mice reduces PAI‐1 protein in BAL fluid and attenuates bleomycin‐induced lung fibrosis

**Figure 6:**
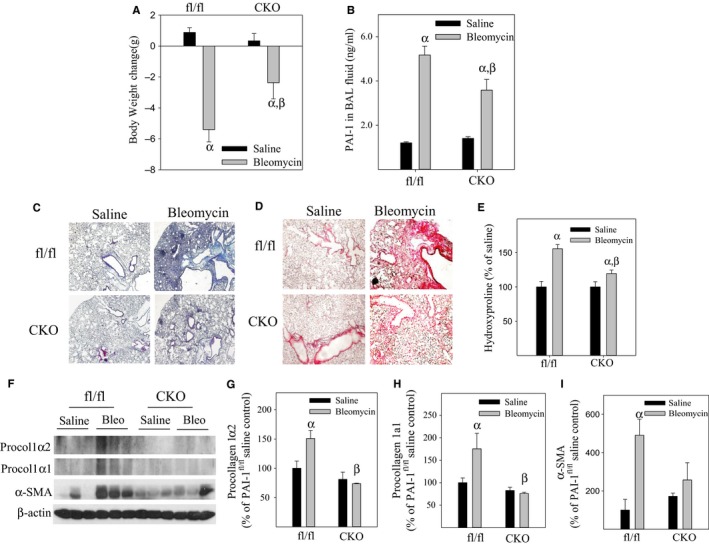
Deletion of the PAI‐1 gene specifically in ATII cells in mice attenuates bleomycin‐induced lung fibrosis. (A) Body weight changes before and 14 days after bleomycin/saline treatment. (B) The amount of PAI‐1 protein in BAL fluid measured by ELISA. (C) Trichrome staining of collagen and (D) Sirius red staining of collagen. (E) Hydroxyproline content in mouse lung measured using the Hydroxyproline Assay Kit (Chrondrex, Inc) and expressed as % of hydroxyproline in saline‐treated fl/fl mice. (F–I) Western analyses of procollagen 1α1, procollagen 1α2, and alpha‐smooth muscle actin (α‐SMA) in mouse lung tissue. α, Significantly different from same genotype, saline‐treated mice; β, significantly different from bleomycin‐treated PAI‐1^fl/fl^ mice (*P* < 0.05, *n* = 3–8).

ATII cells have been shown to play a central role in the development of lung fibrosis (Lijnen, 2005; Chao, 2015; Li & Kurokawa, 2015). To determine whether deletion of PAI‐1 specifically in ATII cells protects mice from bleomycin‐induced lung fibrosis, 8‐ to 10‐week‐old CKO and wild‐type (PAI‐1^fl/fl^) mice were injected with tamoxifen, challenged with 2 U/kg bleomycin, and euthanized 14 days after bleomycin instillation. The results show that specifically knocking out the PAI‐1 gene in ATII cells in mice attenuates bleomycin‐induced body weight loss (Fig. 6A) and PAI‐1 accumulation in bronchoalveolar lavage fluid (BALF) (Fig. 6B), although it has no significant effect on the body weight and BALF PAI‐1 level of unchallenged mice. Deletion of PAI‐1 specifically in ATII cells in mice also attenuates bleomycin‐stimulated increases in collagen deposition (Fig. 6C,D), hydroxyproline accumulation (Fig. 6E), and expression of procollagen 1α2 and procollagen 1α1 as well as alpha‐smooth muscle actin (α‐SMA) (Fig. 6F–I) in mouse lungs. Together, the results suggest that ATII cells are an important source of BALF PAI‐1 in fibrotic lung and that ATII cell senescence contributes importantly to the development of lung fibrosis.

## Discussion

Fibrosis is a common pathological feature of many lung diseases, including IPF, an aging‐related progressive fatal lung disorder with unknown etiology (Maclaine & Hupp, 2011; Mark & Thurlimann, 2012). There is no cure for these fibrotic diseases due to incomplete understanding of the pathogenesis. ATII cells are progenitor cells of the alveolar epithelium; ATII cell senescence is evident in IPF and in experimental lung fibrosis models (Buckbinder *et al*., 1995; Chang *et al*., 2010; Bhandary *et al*., 2013; Barkauskas & Noble, 2014; Chao, 2015; Childs *et al*., 2015). The mechanism underlying ATII cell senescence in fibrotic lung diseases, however, is unknown. Using IPF lung tissues, cultured ATII (L2) cells, and a PAI‐1 conditional knockout mouse model, we show, for the first time in this study, that PAI‐1 induces and mediates bleomycin‐ and doxorubicin‐induced ATII cell senescence *in vitro* and *in vivo*. Our data also show that suppression of bleomycin‐induced ATII cell senescence in mice by specifically knocking out the PAI‐1 gene in these cells is associated with attenuation of lung fibrosis. These results suggest that elevation of PAI‐1 contributes importantly to ATII cell senescence in fibrotic lung diseases.

Although PAI‐1 has been shown to induce cell senescence in different types of cells (Fernandez Perez *et al*., 2010; Eren *et al*., 2014a,b; Ghosh *et al*., 2016), the molecular mechanism whereby PAI‐1 promotes cell senescence remains poorly understood. p53 plays a central role in the induction of cellular senescence (Marudamuthu *et al*., 2015), mainly through inducing p21, an inhibitor of cyclin‐dependent kinases (CDKs), leading to dephosphorylation and activation of cell cycle repressor retinoblastoma (Rb). In this study, we show, for the first time, that PAI‐1 protein increases, whereas PAI‐1 siRNA/shRNA and PAI‐1 inhibitor TM5275 suppress bleomycin‐ and/or doxorubicin‐induced, p53 and p21 expressions as well as SA‐β‐gal activity in cultured ATII (L2) cells and in mouse lung ATII cells *in vivo*. This is associated with a decrease (by PAI‐1 protein or bleomycin) and an increase (by PAI‐1 siRNA/shRNA) in Rb phosphorylation, respectively. Silencing p53 in L2 cells, on the other hand, dramatically reduces PAI‐1 protein‐induced p21 expression and L2 cell senescence. Our data strongly suggest that PAI‐1 induces ATII cell senescence, at least in part, through increasing p53 expression and activating p53‐p21‐Rb cell cycle repression pathway. This is first report showing that PAI‐1 positively regulates p53 expression in epithelial cells. We want to stress that the effects of PAI‐1 on p53 expression seem to be cell type specific (Disayabutr *et al*., 2016; Ghosh *et al*., 2016). We showed in a previous study that a small molecule PAI‐1 inhibitor TM5275 induced p53 in fibroblasts (Disayabutr *et al*., 2016). A recent study from a different laboratory demonstrated, on the other hand, that PAI‐1 inhibitor TM5441 suppressed doxorubicin‐induced p53 expression in endothelial cells (Ghosh *et al*., 2016). The results from this study further confirm the complexity of cell type‐dependent regulation of p53 expression by PAI‐1.

The mechanism whereby PAI‐1 increases p53 protein in ATII cells is currently unknown. Posttranslational modifications, including phosphorylation and ubiquitination, play a critical role in p53 protein stability and transactivation function (Huang *et al*., 2012; Marzec *et al*., 2015). Phosphorylation of p53 at serine 15 and serine 20 (serine 18 and serine‐23 in rodents) prevents the binding of p53 to murine double minute 2 (MDM2), a major E3 ubiquitin ligase involved in p53 degradation, and thereby stabilizes p53 protein (Huang *et al*., 2012, 2015). Our studies show that treatment of rat ATII (L2) cells with PAI‐1 protein increases, whereas silencing PAI‐1 with PAI‐1 shRNA or inhibition of PAI‐1 activity with a small molecule PAI‐1 inhibitor TM5275 decreases, p53 phosphorylation at serine‐18 residue, suggesting that PAI‐1 increases p53 protein probably by increasing p53 serine 15 and/or serine 20 phosphorylation and thereby suppressing its degradation. More studies are needed to determine the molecular mechanism whereby PAI‐1 suppresses p53 phosphorylation and potentially degradation in these cells.

It should be stressed that p53 has been shown to regulate PAI‐1 expression in different types of cells (Osterholzer *et al*., 2011; Nevadunsky *et al*., 2013; Mohapatra *et al*., 2016). It has also be reported that PAI‐1 functions downstream of p53 in replicative senescence of cultured fibroblasts (Eren *et al*., 2014a). In this study, we show that silencing p53 with p53 siRNA reduces the basal level of PAI‐1 and attenuates PAI‐1 protein‐induced PAI‐1 expression in L2 cells (Fig. 4), further supporting the notion that p53 regulates PAI‐1 expression. However, using both pharmacological and genetic approaches, we show in this study that PAI‐1 induces p53 and activates p53/p21/Rb pathways in ATII cells *in vitro* and *in vivo*. Silencing p53 almost completely abolishes PAI‐1 protein‐induced p21 expression and L2 cell senescence. These data strongly suggest that PAI‐1 induces p53 and that p53 functions downstream of PAI‐1 in the induction of ATII cell senescence. We also want to mention that, besides inducing senescence, PAI‐1 also plays a role in apoptosis. Although increased PAI‐1 has been shown in almost all types of senescent cells studied, effects of PAI‐1 on apoptosis sensitivity are cell type‐dependent. It has been reported that increased PAI‐1 expression is associated with increased sensitivity of ATII cells to apoptosis (Osterholzer *et al*., 2011; Hogan *et al*., 2014; Schafer *et al*., 2017) but increased apoptosis resistance in fibroblasts (El‐Deiry *et al*., 1992; Serrano‐Mollar *et al*., 2007; Citrin *et al*., 2013; Disayabutr *et al*., 2016). More studies are needed to understand the mechanism whereby PAI‐1 differentially regulates p53 expression and apoptosis sensitivity in different types of cells.

Insulin‐like growth factor binding protein 3 (IGFBP3) is a major insulin growth factor (IGF) binding protein, which binds to IGF and inhibits its signaling. IGFBP3 expression is increased in senescent cells and is believed to mediate cell senescence in different types of cells (Fernandez Perez *et al*., 2010; Eren *et al*., 2014b; Ghosh *et al*., 2016). Interestingly, it has been reported that PAI‐1 promotes senescence in MCF‐7 breast cancer cells by inhibiting tPA‐mediated proteolysis of IGFBP3 (Eren *et al*., 2014b). Increased PAI‐1 expression has also been shown to be responsible for the increase in IGFBP3 expression, cell senescence, and lifespan shortening in *Klotho*‐deficient (*kl/kl*) mice, a murine aging model (Fernandez Perez *et al*., 2010). Consensus sites for p53 binding have been identified in intronic regions of the IGFBP3 gene and wild‐type p53 increases IGFBP3 expression in response to DNA damage (Shetty *et al*., 2008, 2012; Sisson *et al*., 2010). These results suggest that IGFBP3 may functions downstream of PAI‐1 and p53 in the induction of senescence. Whether PAI‐1 induces IGFBP3 in ATII cells and whether PAI‐1 induces ATII cell senescence by increasing p53 and thereby IGFBP3 remains to be determined.

PAI‐1 expression is increased in lung fibrotic diseases including IPF and in experimental lung fibrosis models (El‐Deiry *et al*., 1992; Dimri *et al*., 1995; Citrin *et al*., 2013; Disayabutr *et al*., 2016). Which types of cells are the major contributors of PAI‐1 in fibrotic lungs and how PAI‐1 promotes lung fibrosis, however, remain to be determined. It has been reported that, in diphtheria toxin (DT)‐induced ATII cell injury and fibrosis model, ATII cells and macrophages are the major contributors of PAI‐1 in the alveolar compartment (Zappa *et al*., 2009). Increased PAI‐1 expression contributed to DT‐induced ATII cell injury and lung fibrosis as knockout of the PAI‐1 gene attenuated DT‐induced ATII cell injury and lung fibrosis (Zappa *et al*., 2009). Using tamoxifen‐inducible ATII cell‐specific PAI‐1 knockout mouse model, we show, in this study, that knockout of the PAI‐1 gene specifically in ATII cells in mice significantly attenuates bleomycin‐stimulated increase in BALF PAI‐1 protein, although it has no significant effect on the basal level of PAI‐1 in BALF. Our results also show that deletion of the PAI‐1 gene in ATII cells alone significantly reduces bleomycin‐induced accumulation of collagen and hydroxyproline in mouse lung. These data suggest that ATII cells are important source of BALF PAI‐1 in fibrotic lung, although they are not the major contributors of BALF PAI‐1 under unchallenged/normal condition. These data also suggest that ATII cell PAI‐1 plays a pivotal role in the development of lung fibrosis and that PAI‐1 promotes lung fibrosis in part by inducing ATII cell senescence. Further studies are warranted to determine how senescent ATII cells contribute to the development of lung fibrosis and the role of PAI‐1 in this process.

In summary, we demonstrate here, for the first time, that PAI‐1 induces p53, activates p53‐p21‐Rb cell cycle repression pathway, and promotes senescence in ATII cells *in vitro* and *in vivo*. These findings may have a significant impact on the research beyond lung fibrosis as PAI‐1 expression is increased with age and in many aging‐related pathological conditions.

## Experimental procedures

### Generation of PAI‐1 conditional knockout mice

See supplementary material (Fig. S1, Supporting information) for the details. Western blotting and immunofluorescence staining techniques were used to confirm Tmx‐inducible PAI‐1 conditional knockout phenotype in isolated ATII cells after mice were injected with tamoxifen or oil (Fig. S2, Supporting information). All procedures involving animals were approved by the Institutional Animal Care and Use Committees at the University of Alabama at Birmingham and conducted at the UAB animal facilities under specific pathogen‐free conditions.

### Cell culture and treatment

L2 cells, originally derived from type II pneumocytes of adult rat lungs, were obtained from the American Type Culture Collection (Rockville, MD) and cultured with Ham's F‐12 medium supplemented with 10% fetal bovine serum, 100 units/mL penicillin, and 100 μg/mL streptomycin at 5% CO_2_ and 37 °C.

### Establishment of PAI‐1 knockdown ATII cell line

L2 cells were stably transduced with nontarget shRNA retrovirus or PAI‐1 shRNA retrovirus (Origene, Cat No TR30013 and TF709263, respectively) according to the manufacturer's instructions. Briefly, retrovirus vector pGFP‐V‐RS‐Control shRNA or pGFP‐V‐RS‐rat PAI‐1 shRNA was transfected into Phoenix‐AMPHO retrovirus package cells. The supernatant containing the viruses was harvested 48 h posttransfection to infect L2 cells. At 24 h postinfection, transduced cells were selected with 1 μg/mL puromycin for 1 week. PAI‐1 protein was determined by Western blotting to confirm the knockdown phenotype.

### Induction of lung fibrosis and collection of samples

Eight‐ to 10‐week‐old male PAI‐1^fl/fl^ (WT control) and Sftpc‐CreER:PAI‐1^fl/fl^ mice were intraperitoneally injected with 100 mg/kg Tmx for 7 days and then challenged with 2 U/kg of bleomycin (dissolved in saline) or saline alone through oropharyngeal instillation. Mice were euthanized 14 days after challenge. Bronchoalveolar lavage (BAL) was performed and pulmonary artery vascular beds perfused as we have described previously (El‐Deiry *et al*., 1992; Disayabutr *et al*., 2016). Left lung was then fixed with 10% PBS‐buffered formalin and the rest of the lung frozen immediately in liquid nitrogen for biochemistry analyses as we have described previously (Disayabutr *et al*., 2016).

### Isolation of ATII cells from mouse lung

Mouse lung ATII cells were isolated following the protocol described previously (Zhang *et al*., 2012) with a few modifications. Briefly, mouse lungs were instilled with 2 mL protease solution (300 U/mL collagenase type I, 4 U/mL elastase, 5 U/mL dispase, and 100 μg/mL DNase I in HBSS), minced by razor, and incubated at 37 °C for 25 min. Digestion was stopped with 50% DMEM/50% F12 containing 3% fetal bovine serum and suspension washed in HBSS and then incubated with 2 mL HBSS containing 0.1% trypsin–EDTA, 100 μg/mL DNase I for 20 min at 37 °C. Following tissue dissociation, cell suspensions were filtered through a 40‐μm nylon mesh, washed, and treated with ACK (150 mm NH4Cl, 10 mm KHCO3, 0.1 mm EDTA) solution to lyse red blood cells and then suspended in DMEM/F12 medium containing 1% fetal bovine serum. Macrophages and lymphocytes were removed by incubation with biotinylated rat anti‐mouse CD45 and rat anti‐mouse CD16/32. (BD Biosciences). The cells were then cultured in DMEM/F‐12 medium containing 10% FBS in 100‐mm culture dishes at 37°C for 2 h to remove lung fibroblasts. The suspended ATII cells were harvested for further analysis. Immunostaining with anti‐SPC antibody confirms that >90% cells are ATII cells.

### Immunofluorescence staining

To reveal PAI‐1, p53, and p21 proteins in mouse lung tissue, double immunostaining was conducted using formalin‐fixed, paraffin‐embedded tissue slides with antibodies to mouse PAI‐1 (Molecular Innovations, Cat No MA‐33H1F7), p53 (Ancell, Cat No 227‐020), or p21 (Santa Cruz, Cat No SC‐6246) and rabbit polyclonal anti‐mouse proSP‐C antibody (Millipore, Cat No AB3786), following the protocol as we have described previously (El‐Deiry *et al*., 1992). More than 300 ATII cells were counted in nine different areas per mouse lung; PAI‐1‐, p53‐, or p21‐positive ATII cells are expressed as percentage of total ATII cells.

For immunofluorescence staining of primary mouse ATII cells, 2 × 10^5^ isolated ATII cells were seeded onto 35‐mm culture dish with glass cover slip coated with rat collagen I for 24 h. Cells were fixed by 4% paraformaldehyde and incubated with anti‐PAI‐1 and p53 antibodies or anti‐p21 and anti‐SPC antibodies following the similar protocol as described above. More than 300 ATII cells were counted per mouse and p21‐positive ATII cells (SPC positive) as well as PAI‐1 and p53 double‐positive cells were expressed as % of total ATII cells. To reveal cell proliferation, 2 × 10^5^ L2 cells, cells were fixed, permeabilized, and then incubated with rabbit polyclonal antiproliferating cell nuclear antigen (PCNA, Cell Signaling, Beverly, MA, USA Cat No 13110S) followed by fluorescein‐conjugated anti‐rabbit antibody (Vector Lab, Burlingame, CA, USA Cat No FI‐1000, green color). The nuclei were visualized by DAPI staining.

### Measurement of the activity of senescence associated beta‐galactosidase (SA‐β‐gal)

The activity of SA‐β‐gal in cultured L2 cells and in freshly isolated mouse ATII cells was determined using 5‐bromo‐4‐chloro‐3‐indolyl P3‐D‐galactoside (X‐gal), following the protocol described previously (Zhang *et al*., 2013). For mouse ATII cells, freshly isolated cells were span onto slides using a Statspin Cytofuge, fixed, and stained with X‐gal immediately after isolation. SA‐β‐gal‐positive cells (blue color) were counted under microscope and expressed as % of total cells.

### Western blot analysis

Cells were lysed in cell lysis buffer, and lung tissues were homogenized in 0.25 m sucrose buffer containing protease inhibitor (Sigma, St. Louis, MO, USA P8340) and phosphatase inhibitor cocktails (Sigma, P5726) and centrifuged at 3000 *g,* 4°C, for 10 min and then in 100 000 *g* for 60 min. Westerns were conducted with supernatants as we have described previously (El‐Deiry *et al*., 1992; Disayabutr *et al*., 2016) with the following antibodies: PAI‐1 (Molecular Innovation, Novi, MI, USA ASMPAI‐GF, ASRPAI‐GF), α‐SMA (Biocare, CM001B), p53 (Santa Cruz, SC‐6243), p21 (Santa Cruz, Dallas, TX, USA SC‐397), procollagen 1α1 (Santa Cruz, SC‐8784‐R), procollagen 1α2 (Santa Cruz, SC‐8788), and β‐actin (Sigma, A5441). The protein bands were visualized using the ECL detection system (Amersham, Piscataway, NY, USA), semi‐quantified using ImageJ software, and normalized by β‐actin band intensity.

### ELISA of PAI‐1 protein in bronchoalveolar lavage fluid (BALF)

PAI‐1 protein in mouse BALF was determined by ELISA as we have described previously (Disayabutr *et al*., 2016).

### Trichrome and Sirius red staining of collagens in mouse lung tissue

Trichrome staining was conducted as we have described previously (Disayabutr *et al*., 2016), whereas Sirius red staining performed following the protocol described by others (Zuckerman *et al*., 2009).

### Hydroxyproline measurement

Hydroxyproline content in the right lungs of mice was measured using the Hydroxyproline Assay Kit from Chondrex, Inc (catalog number: 6017), according to the protocol provided by the manufactory. The results were calculated based on the standard curves derived from 4‐hydroxy‐L‐proline.

### Statistical analysis

Data were evaluated by one‐way ANOVA. Statistical significance was determined post hoc by Tukey's test.

## Funding

This work is supported by National Heart, Lung, and Blood Institute to Rui‐Ming Liu (5R01HL088141; R56HL131054) and to Victor J. Thannickal (P01 HL114470).

## Author's contributions

CJ conducted the experiments and analyzed and wrote the manuscript; TL helped with alveolar type II cell isolation; GL, VA, YZ, and ABC contributed intellectually to the experimental design and edited the manuscript; VJT contributed data interpretation and manuscript writing; RML conceived the project, designed the experiments, and wrote the manuscript.

## Conflict of interest

The authors have no conflict of interest to declare.

## Supporting information


**Fig. S1** A schematic flow chart of the processes to generate tamoxifen inducible ATII cell specific PAI‐1 conditional knockout mice.
**Fig. S2** Assessment of PAI‐1 gene knockout phenotype in Sftpc‐CreER:PAI‐1^fl/fl^ mice.
