# Supplementary figures and images for: Serpine 1 induces alveolar type II cell senescence through activating p53‐p21‐Rb pathway in fibrotic lung disease
Source: Aging Cell. 2017 Jul 19;16(5):1114–24. doi: 10.1111/acel.12643 (PMC5595683; doi:10.1111/acel.12643)

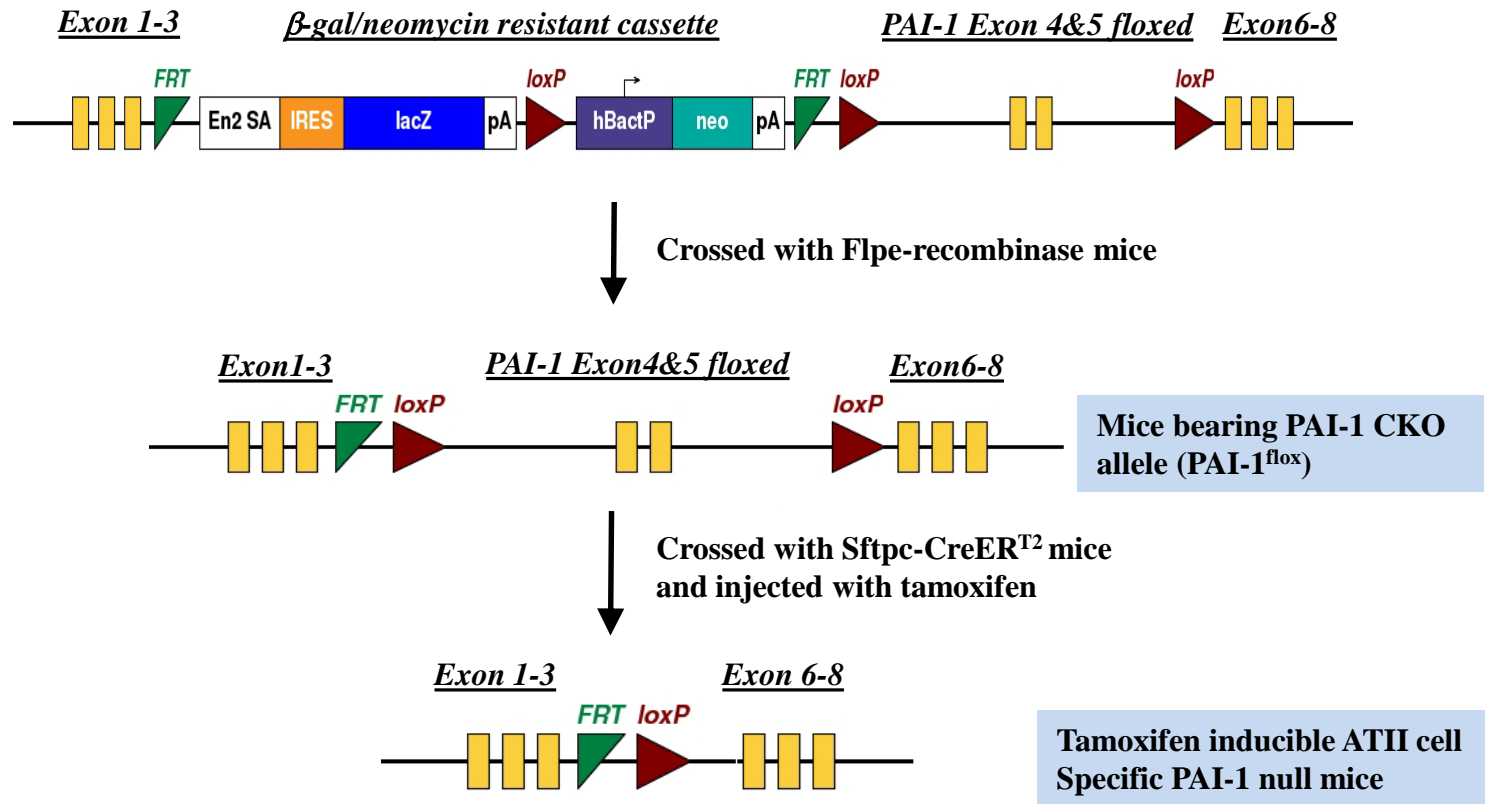

Figure S1

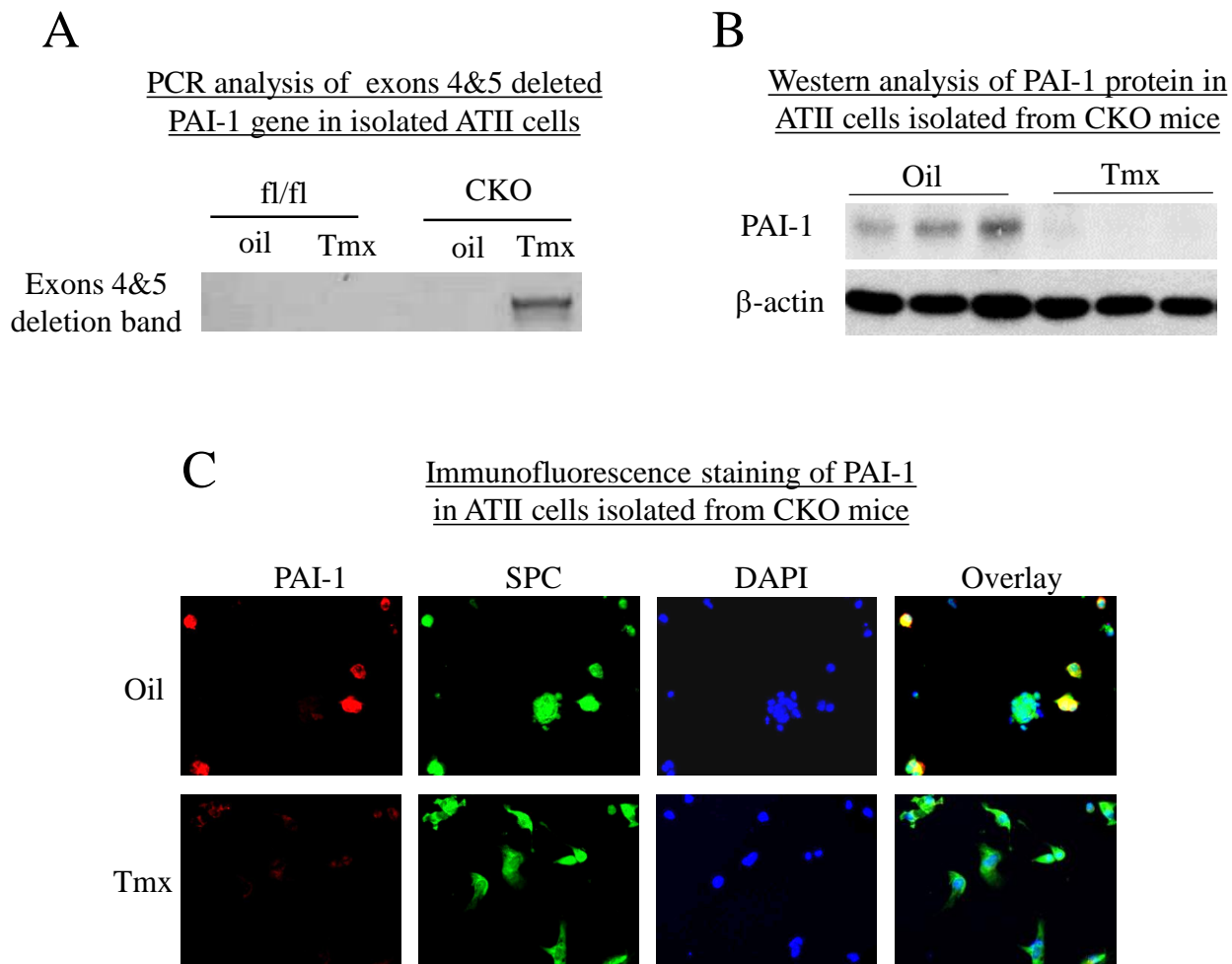

Figure S2

Supplement: Supplementary file 1 — Fig. S1 A schematic flow chart of the processes to generate tamoxifen inducible ATII cell specific PAI‐1 conditional knockout mice. Fig. S2 Assessment of PAI‐1 gene knockout phenotype in Sftpc‐CreER:PAI‐1fl/fl mice. [file ACEL-16-1114-s001.pdf]
