# Supplementary material for: Serpine 1 induces alveolar type II cell senescence through activating p53‐p21‐Rb pathway in fibrotic lung disease
Source: Aging Cell. 2017 Jul 19;16(5):1114–24. doi: 10.1111/acel.12643 (PMC5595683; doi:10.1111/acel.12643)
Supplement: Supplementary file 2 [file ACEL-16-1114-s002.docx]

**Figure legends for supplemental data**

**Fig S1. A schematic flow chart of the processes to generate tamoxifen inducible ATII cell specific PAI-1 conditional knockout mice.** Embryonic stem (ES) cells harboring a PAI-1 allele with the exons of 4&5 flanked by 2 lox P sites were injected into blastocysts from C5BL/6j. Male chimeras were crossed to female C57BL/6j mice to establish germline transmission of the PAI-1 floxed allele. Germline transmission mice were subsequently bred to mice expressing Flp-recombinase to excise the β-gal/neomycin resistance cassette for creation of mice bearing the conditional PAI-1 knockout allele (PAI-1^flox^). Homozygous PAI-1^flox^ (PAI-1^fl/fl^) mice were then crossed with tamoxifen (Tmx) inducible surfactant protein C (SP-C) promoter driven Cre recombinase expressing mice (kindly provided by Dr. Brigid M. Hogan, Duke university) to generate tamoxifen inducible ATII cell specific PAI-1 conditional knockout (Sftpc-CreER:PAI-1^fl/fl^) mouse model.

**Fig S2. Assessment of PAI-1 gene knockout phenotype in Sftpc-CreER:PAI-1^fl/fl^ mice.** Eight to ten weeks old Sftpc-CreER:PAI-1^fl/fl^ and PAI-1 floxed (PAI-1^fl/fl^) mice (Sftpc-CreER negative littermates) were intraperitoneally injected with tamoxifen (100 mg/kg, dissolved in corn oil) or corn oil for 7 consecutive days. ATII cells were isolated and PCR was performed with isolated DNA to check the specific deletion band of the PAI-1 gene, whereas PAI-1 protein assessed by immunofluorescence staining and by Western blotting. **A)** PCR analysis of the exon 4&5 deletion band in the PAI-1 gene in ATII cells. **B)** Western analyses of PAI-1 protein in ATII cells. **C)** Immunofluorescence staining of PAI-1 protein in ATII cells (>95% of isolated cells are ATII cells by SPC staining).
